# Smartcard: an integrated approach for contaminant monitoring, from field to laboratory

**DOI:** 10.1007/s00216-024-05626-w

**Published:** 2024-11-12

**Authors:** Ariadni Geballa-Koukoula, Linda Willemsen, Erik Beij, Richard van Hoof, Alexander Elferink, Khalil Geballa-Koukoulas, Jeroen Peters, Marco H. Blokland, Gert IJ. Salentijn

**Affiliations:** 1 Wageningen Food Safety Research, Wageningen University and Research, P.O. Box 230, 6700 AE Wageningen, The Netherlands; 2 Laboratory of General and Agricultural Microbiology, Department of Crop Science, Agricultural University of Athens, Iera Odos 75, 118 55 Athens, Greece; 3 Laboratory of Organic Chemistry, Wageningen University, Stippeneng 4, 6708 WE Wageningen, The Netherlands

**Keywords:** On-site application, Dried blood spot (DBS) card, Lateral flow immunoassay (LFIA), Fipronil

## Abstract

Effective food safety monitoring requires a multi-step approach from farm to fork, involving different methods, ranging from convenient screening devices to sophisticated laboratory confirmatory testing. However, sample transportation to routine laboratories is time-consuming and expensive. Simplified on-site sampling followed by laboratory analysis offers a potential solution. Dried blood spot (DBS) cards ensure stability and ease of sample transportation and are used in clinical testing. However, the applicability of such an approach could be broader and include the storage of dried extract from more complex (solid) matrices. Therefore, a simplified approach is presented here, using DBS cards for on-site sampling and subsequent laboratory confirmation for food contaminants. To achieve this, an analytical tool (Smartcard) was designed using 3D-printing technology. As a proof of concept, the approach was applied to detect the pesticide fipronil, which is widely used in ornamental flower production to limit pests and on poultry farms. The Smartcard can securely store the sample extracts on a DBS card (dried extract spot (DExS) card), incorporate the lateral flow immunoassay (LFIA) and immediately provide an estimate of contamination levels. After simplified in-syringe extraction of the sample, the LFIA allows direct screening of fipronil (half maximum inhibitory concentration of 6.5 µg/l with calibration standards), and the same sample extract can be directly applied to the DExS card for storage and transport to the laboratory, where analyte re-extraction and instrumental analysis is performed using ultra high-pressure liquid chromatography tandem mass spectrometry (UHPLC-MS/MS) detecting fipronil down to 0.8 µg/kg.

**Graphical Abstract:**

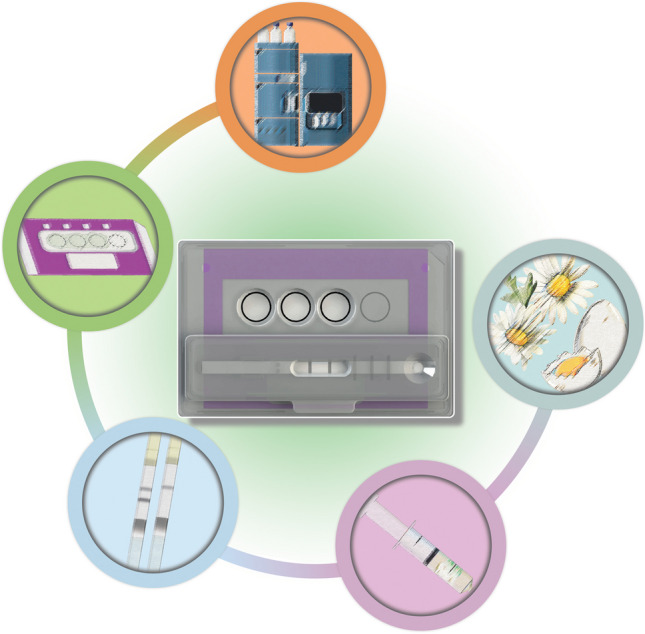

**Supplementary Information:**

The online version contains supplementary material available at 10.1007/s00216-024-05626-w.

## Introduction

Regulatory bodies set maximum levels for many contaminants in legislation; therefore, constant monitoring for contaminants in food commodities is crucial to ensure food safety [1, 2]. Current food safety monitoring involves a reliable and sophisticated, yet complex, and time-consuming approach, starting from sample collection, transportation, storage and pre-treatment, leading to instrumental analysis [3]. Moreover, this approach is costly and restricted to well-equipped laboratories. To circumvent this traditional route and these challenges, on-site analysis using appropriate test kits and simplified on-site sampling and extraction, followed by laboratory analysis, have emerged as potential alternatives for a rapid food safety assessment [4]. While on-site analytical approaches offer convenience [5, 6], their performance is not sufficient or suited for confirmatory analysis, in terms of sensitivity, accuracy and food safety-related identification criteria [7]. Thus, in practice, an approach is to use on-site screening, for example, for risk-based sampling and/or reducing the number of samples sent to the laboratory by eliminating compliant samples early on; in doing so, only suspect samples are transported and submitted for further confirmatory analysis. Still, in that workflow, the entire sample is transported and re-extracted in the laboratory before analysis, duplicating prior efforts. Therefore, integrating simplified on-site sampling, and storing the sample in a format that can be analysed directly in the laboratory, offers an attractive next step in streamlining this process. Efforts are being made to develop suitable on-site sampling devices [8, 9] with one promising approach being the utilization of dried blood spot (DBS) cards [10, 11].

DBS sampling has a long history dating more than half a century ago when it was first introduced for phenylketonuria determination in newborns’ blood samples [12]. Ever since, DBS sampling has revolutionized clinical testing, particularly in prenatal care [13, 14]. DBS cards allow for the collection of low-volume samples by applying them to cellulose paper, eliminating the need for intricate laboratory equipment for sample pre-treatment and enabling easy transportation and storage due to the stability of the card material and the stability of the dried analyte on the card [10, 15]. The essential features of DBS sampling, including its simplicity of handling, accessibility and cost-effectiveness, serve as fundamental requirements for any on-site sampling method. By incorporating these features, non-specialized or minimally trained individuals can utilize on-site sampling methods, facilitating widespread adoption and contributing to efficient (bio)monitoring. While DBS cards have primarily been associated with blood, plasma sampling and maternal milk, their potential for sample extracts storage remains underexplored [16, 17]. Here, we postulate that the steps undertaken for carrying out on-site screening assays, for example, using a lateral flow immunoassay (LFIA), can be leveraged to simplify the remainder of the analytical workflow, allowing laboratory confirmation at reduced costs and efforts. A key component of our approach is the use of 3D-printing technology [18, 19] to create a customized cartridge, referred to as a Smartcard. The Smartcard serves a dual purpose. First, it serves as an LFIA cassette for rapid on-site screening of a specific targeted compound. Second, it securely stores sample extracts made for the LFIA, facilitating the sample extract transportation. These dried extract spot (DExS) cards can be (re-)extracted in the lab for (ultra) high-pressure liquid chromatography-tandem mass spectrometry ((U)HPLC–MS/MS) for identification and quantification of the previously screened contaminants.

One example from the food safety field that is expected to benefit from combining quick screening with risk-based on-site sampling, facilitating more economical use of instrumental analysis, is the monitoring of the insecticide fipronil. Fipronil, a phenylpyrazole-class insecticide, is utilized to combat the red mite *Dermanyssus gallinae* in poultry production and veterinary products [20].

Due to its high physical stability and slow degradation, the consumption of fipronil-contaminated commodities results in its accumulation and extensive metabolism (oxidation) in the liver, which allows it to be distributed throughout the body [21, 22]. Inside the body, fipronil will bind to γ-aminobutyric acid (GABA) receptors and glutamate-gated chloride channels, leading to nerve cell excitation. Additionally, fipronil consumption can lead to side effects varying from gastrointestinal issues to hormone disbalance and thyroid disruption [23, 24]. A maximum residue limit of 5 µg/kg for total fipronil including metabolites in chicken meat and eggs has been set in the European Union (EU) [25]. Despite this regulatory limit, fipronil exceedances have been reported in poultry production. For instance, in 2017, fipronil captivated the public due to a major scandal when contaminated eggs above the regulatory limit were discovered in several EU countries, resulting in enormous food waste and economic losses because of the widespread recall of supplies from the market [26]. Nonetheless, in 2018, a study was conducted on 91 different egg samples from the market, none of which was tested above the regulatory limit of 5 µg/ml [27]. Notably, fipronil is not exclusively used in poultry production but also in treating ornamental flowers to mitigate pest spread and improve product quality [28]. High fipronil levels have been found in tea and edible flowers [29, 30] and fipronil has been linked with mortality of honey bees [31]. The absence of regulations and maximum residue limits for non-consumable flowers contributes to this practice, and raises questions of whether indirect exposure could be a risk that needs to be mitigated in the future [32–34]. Our study introduces a framework for a combined, efficient on-site screening method followed by instrumental analysis to monitor fipronil residues. The developed sampling and analysis approach holds promise for rapid screening/risk-based sampling and subsequent laboratory confirmation of contamination, contributing to improved food safety measures. The extension to other small molecule contaminants is foreseen.

## Material and methods

### Reagents and consumables

Acetonitrile, methanol, acetone and ethyl acetate of analytical purity grade and formic acid 98% v/v and a fipronil standard solution of 2 mg/ml in methanol were purchased from Merck (Darmstadt, Germany). Milli-Q water (conductivity of 18.3 MΩ/cm) was obtained from a water purification system from Merck (Darmstadt, Germany). Wild ornamental flowers (*Leucanthemum vulgare*) were manually collected from an area in Wageningen, The Netherlands, and biological eggs were purchased from a local supermarket. Both flowers and eggs were used for the experiments as blanks, as confirmed by UHPLC-MS/MS analysis. Homogenization of the spiked egg yolk was achieved with a mortar and pestle.

For the LFIA, a fipronil-specific monoclonal antibody (anti-fip mAb) (8054, 4.8 mg/ml) and a fipronil conjugate with bovine serum albumin (fip-BSA) (8054, 3.6 mg/ml) were purchased from Ecalbio (Wuhan, China). The species-specific polyclonal antibodies, i.e. goat anti-mouse IgG FcY (GaM) (code 115–005-071, 1.8 mg/ml) and donkey anti-goat IgG (DaG) polyclonal (H + L) antibodies (code 705–005-003, 1.3 mg/ml) were obtained from Jackson ImmunoResearch (Sanbio, Uden, The Netherlands). The running buffer for the LFIA was PBS-TB, a 0.01 M phosphate-buffered saline (PBS) solution containing 0.05% v/v Tween-20 and 1% w/v bovine serum albumin (BSA). The PBS stock solution was prepared by dissolving one PBS buffer tablet (Merck, Darmstadt, Germany) in 1 l of Milli-Q water, resulting in a solution with a composition of 140 mM sodium chloride (NaCl), 10 mM phosphate buffer and 3 mM potassium chloride (KCl), pH 7.4 at 25 °C. Tween-20 and BSA from Sigma-Aldrich (Zwijndrecht, The Netherlands) were added to achieve a final concentration of 0.05% v/v and 1% w/v, respectively. A spraying buffer of 1 × PBS with 2% w/v BSA and 5% trehalose was also prepared. Amorphous carbon nanoparticles (CNPs) were obtained from Orion Engineered Carbons, Houston, TX, USA.

For the DExS card preparation, DBS cards were purchased from Ahlstrom (Helsinki, Finland), and paraffin wax was purchased from Merck (Darmstadt, Germany).

For the UHPLC-MS/MS, a solution of 5 mM ammonium formate (Merck, Darmstadt, Germany) was prepared in Milli-Q water, and 2 ml total recovery vials (Thermo Fisher Scientific, MA, USA) were used for the analysis.

### Extraction solvent selection

The compatibility of the different components of the Smartcard with different types of potential organic extraction solvents, namely methanol, acetonitrile, acetone, ethyl acetate, ethanol and acetonitrile/formic acid 1% v/v, was assessed. For the 3D-printing resin, 3D-printed cubes of 450 mm^3^ size were immersed in 4 ml organic solvent for 30 min and weighed before and after soaking. The results were assessed as the %difference in weight. The compatibility of the fipronil LFIA (see Extraction solvent selection) for different types of organic solvents was tested by developing the LFIA in a 96-well plate containing GaM-CNP conjugate (1 µl) with 5%, 10% and 20% v/v organic solvent in running buffer. After 10 min of development, the LFIAs were assessed visually for the formation of both test and control lines.

### Fipronil extraction

#### Fipronil extraction from samples of eggs

Different extraction times were tested after selecting the optimal extraction solvent (methanol) to determine the shortest extraction time. The extraction time of fipronil from eggs was evaluated by analysing homogenized spiked egg yolk and comparing the UHPLC-MS/MS area signal with fipronil standard solutions. Spiked egg yolk with 5 µg/kg fipronil was incubated in a 1:1, 1:2 or 1:3 v/v ratio with methanol for 2 min, 5 min, 10 min, 20 min or 30 min. The complete optimized protocol for the extraction of fipronil from eggs consisted of the following: the egg was cracked, and the egg white was discarded, keeping only the egg yolk. One ml of yolk was retrieved with a standard 5-ml syringe, followed by the addition of 1 ml of methanol. The syringe plunger was moved up and down at least ten times to mix the two liquids, and a stopper was positioned in the opening of the syringe. The syringe was then placed on a benchtop Eppendorf ThermoMixer C apparatus (Eppendorf SE, Hamburg, Germany) at 1000 rpm for 20 min and mixed to achieve extraction of fipronil. After 20 min, the stopper was removed, and a Whatman 0.45-µm syringe filter was positioned in the opening of the syringe to retrieve the fipronil extract.

#### Fipronil extraction from samples of flowers

Similarly to the egg yolk, different extraction times were tested for optimal extraction of fipronil from ornamental flowers. Fipronil-spiked samples were made by depositing 5 µg/kg fipronil standard solution on a weighed amount (1 g) of flower and leaves, which was allowed to air dry overnight. Next, the spiked wild ornamental flowers mixed sample (petals, leaves and stems) were incubated in a 1:1 w/v ratio with methanol for 2 min, 5 min, 10 min, 20 min or 30 min. The extraction time of fipronil from flowers was evaluated by comparing the UHPLC-MS/MS area signal with that of fipronil standard solutions. The optimized protocol for extracting fipronil from ornamental flowers consisted of the following steps: first, 1 g of wild ornamental flowers (petals, leaves and stems) was weighed in a 5-ml plastic syringe and used as blank or spiked with fipronil. Then, 1 ml of methanol was drawn up in the syringe, and the plunger was pushed up and down at least 10 times to mix the flowers and the extraction solvent. A stopper was then positioned at the opening of the syringe, and the extraction was performed on a benchtop thermomixer at 1000 rpm for 5 min.

### Smartcard design and fabrication

All the 3D-printed components were designed using the computer-aided design (CAD) software SolidWorks 2021 (Dassault Systèmes SolidWorks Corporation, Waltham, MA, USA) and printed on a high-resolution stereolithography (SLA) printer Form3 (Formlabs, Somerville, MA, USA) at a layer resolution of 100 µm, using Formlabs clear resin (type v4).

### Screening LFIA for fipronil

#### Optimization and preparation

The indirect competitive LFIA (icLFIA) format was chosen for the LFIA construction. The LFIA components included a Unisart CN 95 nitrocellulose membrane (Sartorius, Gottingen, Germany), a glass fibre pad (8951, Ahlstrom, Finland), a sample pad (1660, Ahlstrom, Finland), an absorbent pad (222, Ahlstrom, Finland) and a plastic backing support (Kenosha, Amstelveen, The Netherlands). After assembly, the LFIAs were cut to the desired size using a CM4000 BioDot Guillotine (BioDot Inc., Irvine, CA, USA) and stored at room temperature in aluminium pouches with MiniPax absorbent packets (Merck, Darmstadt, Germany). GaM-CNPs were prepared according to a standard laboratory protocol previously reported [35].

Depending on the experimental stage, the LFIA was developed either in a Cellstar 96-well plate (Greiner Bio-One, Alphen aan den Rijn, The Netherlands) or in a custom-made 3D-printed LFIA cassette. The concentration of the biomolecules of the fipronil LFIA was optimized by manually spotting 0.5 µl fip-BSA and DaG pAb 5 mm apart from each other, on 4-mm-wide blank nitrocellulose membranes using a micropipette. The spotted concentrations of fip-BSA were 0.5 mg/ml and 1 mg/ml, and the DaG pAb was spotted at 0.15 mg/ml, all diluted in 0.01 M PBS. The LFIAs were then developed with different concentrations of the anti-fip mAb. The LFIAs were then dried at room temperature for 30 min and stored in a sealed package with MiniPax absorbent packets. The LFIAs were developed in a 96-well plate containing GaM-CNP conjugate (1 µl), concentration series of the anti-fip mAb (i.e. 19.2 µg/ml, 38.4 µg/ml and 76.2 µg/ml) (1 µl) and fipronil standard at various concentrations (1 µg/l, 10 µg/l, 100 µg/l and 0 µg/l (blank)) in running buffer PBS-TB (98 µl). The spotted LFIAs were placed upright in the well and were assessed visually after 10 min of development.

The optimized LFIA consisted of a fip-BSA test line (*C* = 0.5 mg/ml), while the control line contained species-specific DaG antibodies (*C* = 0.15 mg/ml), both sprayed at a spraying speed of 1 µl/cm. The bioreagents were sprayed on the nitrocellulose membrane using an XYZ 3060 BioDot Dispense Platform (BioDot Inc., Irvine, CA, USA). Details about the LFIA assembly can be retrieved in Supplementary Fig. 1. The fully assembled membranes were cut into LFIAs of 4 mm width.

For LFIA development, 100 µl of either (a) standard solutions of fipronil in running buffer or (b) running buffer/extract (80:20 v/v) was used. Semi-quantitative interpretation of the LFIA visual results was achieved through image acquisition with a Cube analyser (Chembio, Berlin, Germany) after 10 min of LFIA development. Also, corresponding images were captured in ambient lighting using a smartphone (Samsung A50).

#### Sensitivity and preliminary evaluation

The LFIA was assembled with the optimal concentrations of 0.5 mg/ml fip-BSA and 0.15 mg/ml DaG pAb sprayed on the nitrocellulose membrane. The conjugate pad consists of a glass fibre membrane with dried 19.2 µg/ml anti-fip mAb and 10 × diluted GaM-CNP in spraying buffer. The fully assembled LFIAs were placed in a custom-made 3D-printed cassette and developed with calibration standards (1 µg/l, 5 µg/l, 10 µg/l, 100 µg/l and blank, *n* = 2). For the preliminary inter-day variability, the experiment was repeated on three consecutive days in duplicates. In all cases, results were visually assessed (Supplementary Fig. 2) and measured using the Cube Reader 10 min after sample application. The semi-quantitative interpretation was done by calculating the % ratio of test line intensity (*B*) over the control line intensity of the blank (*B*_0_). Based on four-parameter logistic regression analysis, this ratio was plotted against fipronil calibration standards as a dose–response curve.

### DExS card preparation and fipronil re-extraction

A 3D-printed wax stamp was designed consisting of a rectangular base of 42 mm × 17 mm (L × W) with a wall thickness of 1.35 mm and rounded corners (Supplementary Fig. 3). The wax stamp was used to create a wax barrier around the deposition areas on the DBS card, allowing for a larger extract volume to be deposited [36], thus transforming it into the DExS card. To prepare the DExS card, paraffin wax (Merck Darmstadt, Germany) was melted in a beaker at 75 °C. A cellulose pad was soaked with the melted wax. The 3D-printed stamp was then pressed onto the wax-soaked stamp pad for 10 s, to ensure even wax coating on the stamp surface. The DBS card was pre-heated at 80 °C for 1 min and then stamped for 5 s, resulting in the DExS cards.

The recovery of fipronil from the DExS card was assessed by comparing the absolute area intensity of the *m/z* 435.0 > 330.0 transition for fipronil. This comparison was made between UHPLC-MS/MS measurements of a 5 µg/l fipronil standard that had been pipetted onto the centre of the DExS card’s sampling area and at the edge of the sampling area (indicated by a dotted line). Then, a 1-cm-diameter circle cutter (Vaessen Creative, Nuth, The Netherlands) was used to isolate a specified area of the DExS card. The isolated circle was then placed in a total recovery vial, and fipronil was re-extracted with 100 µl methanol, by testing different extraction times, namely 5 min, 10 min, 20 min and 30 min.

The stability of the fipronil on the DExS card was evaluated by depositing 300 µl of 2.5 µg/kg, 5 µg/kg and 7.5 µg/kg fipronil spiked and blank extracts on the DExS card, let to air dry for 30 min, and subsequently housed in a Smartcard at room temperature for a duration of 30 days. Then, the DExS cards were extracted and analysed as described above under optimized conditions.

For the complete Smartcard approach, after extraction of fipronil from the commodities, 300 µl of extract was deposited on the DExS card for lab-based analysis. The DExS card was left to air dry for 1 h, and then each sample circle was cut with a circle cutter and positioned inside a total recovery vial with 100 µl methanol. Fipronil was re-extracted from the DExS by mixing in a benchtop thermomixer for 5 min at 1000 rpm, at room temperature.

### Confirmatory UHPLC-MS/MS analysis

The confirmatory UHPLC-MS/MS analysis was performed on a Xevo TQ-XS tandem triple quadrupole (QqQ) MS system (Waters Corporation, Milford, MA, USA). The UHPLC-MS/MS method was adapted from an in-house confirmatory UHPLC-MS/MS method, and the optimization of the operating conditions was performed with direct injections of standard methanolic solution of fipronil 100 ng/ml with a constant flow of 0.3 ml/min. The optimized conditions for the chromatographic separation were the following: Waters Acquity UPLC HSS T3, 1.8 µm particle size, 2.1 × 100 mm column, 3 µl injection volume, 0.3 ml/min flow rate and isocratic elution using a mobile phase of 5 mM ammonium formate with 0.1% v/v formic acid in Milli-Q water. For the MS detection, electrospray ionization (ESI) was used in negative ionization mode, capillary voltage 2 kV, cone voltage 40 V, source temperature 150 °C, desolvation temperature 300 °C, cone gas N_2_ flow 150 l/h, desolvation gas N_2_ flow 400 l/h, collision gas argon flow 0.15 ml/min, and monitoring of two transitions of *m/z* 435.0 > 330.0 and *m/z* 435.0 > 250.0 in MRM mode, with collision energies 14 eV and 30 eV, respectively. Data was acquired and processed using MassLynx software (Waters). The area ion ratio of the *m/z* 435.0 > 330.0 transition divided by *m/z *435.0 > 250.0 transition was calculated to be 61% (± 8.0) for fipronil. A calibration curve was calculated from the samples analysed using the developed UHPLC-MS/MS method, and the method’s limit of detection (LOD) was estimated by multiplying three times the standard error of the regression (*σ*) divided by the slope of the entire calibration curve (*S*) (LOD = 3 × *σ*/*S*).

For extract analysis, fipronil was re-extracted from the DExS card with 100 µl methanol in a total recovery vial, and the vial was exposed to N_2_ flow in room temperature until total evaporation of the solvent. Finally, the fipronil was reconstituted in 15 µl of methanol for UHPLC-MS/MS analysis.

## Results and discussion

### General concept

**Fig. 1:**
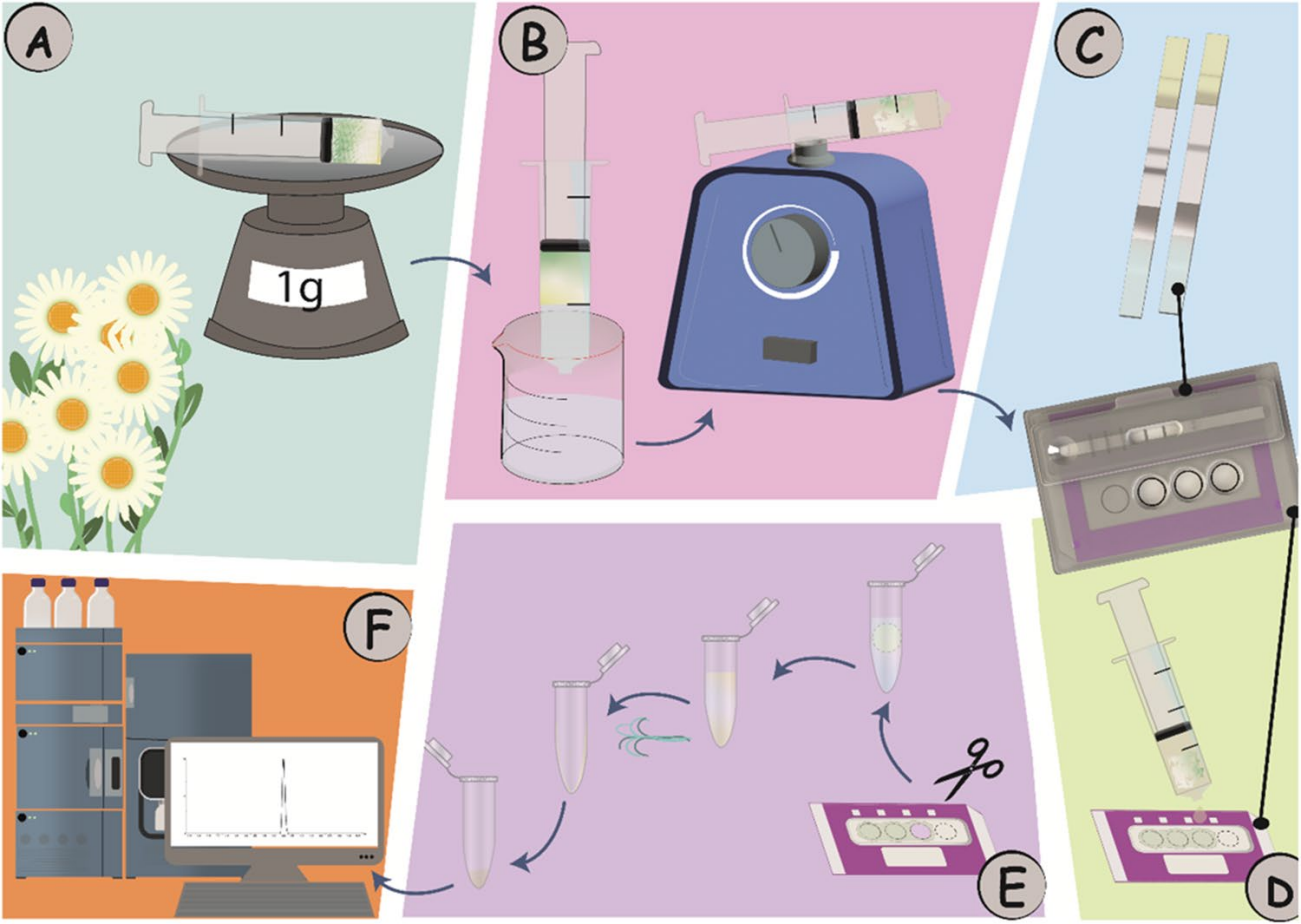
Schematic illustration of the Smartcard approach for fipronil detection from flowers. **A** Weighing of 1 g flower. **B** In-syringe extraction with 1:1 v/w methanol/flower. **C** Development of screening LFIA with diluted extract (20:80 v/v extract/running buffer). **D** Deposition of the methanol extract on the DExS card. **E** Isolation of the extract circle from the DExS card, positioning in a vial, re-extraction of fipronil from the DExS card sample area, evaporation of the methanol in a stream of N_2_ gas and reconstitution with 15 µl methanol. **F** UHPLC-MS/MS confirmatory analysis

We employed a Smartcard, incorporating an LFIA capable of accommodating the DExS card for a simplified on-site screening, sampling and subsequent laboratory confirmation (Fig. 1). The concept was employed for fipronil monitoring in two steps: first, a sample (flower or egg yolk) is extracted, and the sample extract is diluted at a 20:80 ratio with the LFIA running buffer. The diluted extract is used directly to develop an LFIA positioned on the Smartcard. The LFIA provides a binary (yes/no) response with the appearance (or not) of a test line. The Smartcard is designed to accommodate the use of a portable miniaturized camera (Cube Reader) for digital capturing of the LFIA control (C) and test (T) line images, and automated processing of them using an internal software and algorithm for the straightforward semi-quantitative interpretation of the LFIA result [37]. If the result of the screening LFIA is ambiguous (not negative), then the same sample extract is deposited onto the DExS card. The Smartcard securely stores individual DExS cards for transportation to the laboratory, where analyte (re-)extraction and confirmatory UHPLC-MS/MS is conducted to identify and quantify the previously screened contaminant.

### Extraction solvent selection

When performing analyte extraction, it is important to consider several factors, including the compatibility of the extraction solvent with the LFIA components and 3D-printing material, the solubility of the analyte, and its compatibility with the mass spectrometer. According to existing research, fipronil exhibits minimal solubility in deionized water and non-polar organic solvents like toluene, *n*-hexane and 1-octanol. However, it dissolves more effectively in high-polarity organic solvents like methanol, acetone, acetonitrile and ethyl acetate. Acidified organic solvents such as acetonitrile/formic acid 1% v/v have also been effective for dissolving fipronil. Different solvents were tested for their compatibility with the LFIA and/or the 3D-printing polymer (see Supplementary Table 1). The LFIA and 3D-printing polymer are incompatible with polar organic solvents, which can denature antibodies and protein conjugates on the LFIA and potentially warp the 3D-printed casing. While acetone dissolved fipronil effectively, it caused cracking of this 3D-printed cube. Additionally, ethyl acetate and acetonitrile/formic acid 1% v/v, used for fipronil extractions, are incompatible with the LFIA’s bioreagents. It is worth noting, though, that the optimal extraction solvent for fipronil from spiked egg yolk was acetonitrile/formic acid 1% v/v, but the incompatibility of this solvent with the LFIA, causing false positive results, i.e. visual reduction of the test line intensity, does not permit its use in an integrated fashion (see Supplementary Fig. 4). In conclusion, methanol was selected as the most suitable solvent because it did not affect the integrity of the 3D-printing casing and is compatible with the LFIA’s antibodies and conjugates up to a 20% v/v concentration, and fipronil has good solubility in methanol, allowing for further exploration in the extraction recovery from spiked egg yolks and flower matrix.

### Fipronil extraction

#### Fipronil extraction from samples of eggs

The yolk-to-white concentration ratio of fipronil fluctuates during egg development, but the distribution in the egg correlates with the affinity of fipronil and its metabolites for lipids, as well as the fatty acid composition of the egg that contributes to fipronil accumulation in the yolk [20]. Thus, by the end of egg development, the distribution of fipronil in chicken eggs is up to 35 times more favourable for the egg yolk, compared to the egg white [20]. In order to develop a straightforward fipronil extraction from eggs, it is critical to understand the primary materials and methods used and the reasoning behind each selection. The following steps can summarize the process [27, 38–48], which is intricate due to the complexity of the sample. First, the egg sample is homogenized by mixing an appropriate amount of egg yolk and egg whites (typically 5 g). It is worth noticing that although research indicates that fipronil predominantly accumulates in the egg yolk [20], regulation focuses on the analysis of the entire egg [25]. Consequently, using only the egg yolk for fipronil detection could lead to lower detection limits, but might also risk overestimating contamination levels compared to methods that assess the whole egg. So, while the homogenization step might be important for adherence with the regulatory settings, it is omitted due to the localized concentration of fipronil in the yolk and the ease of on-site application for the developed approach. The next step in a typical extraction protocol is the addition of an organic solvent to precipitate the egg proteins and extract the analyte. Fipronil-compatible organic solvents for extraction, such as methanol, ethanol, acetone and ethyl acetate, can be used. A vortex can facilitate faster analyte extraction, while sonication can disrupt the lipid globules that encapsulate fipronil. A centrifuge is also used to aid the rapid sedimentation of the proteins and establish a two-phase separation. A water/salt mixture (e.g. anhydrous magnesium sulfate (MgSO_4_), NaCl, anhydrous sodium citrate (Na_3_Cit) and sodium acetate (NaOAc)) is added to increase the ionic strength of the aqueous phase and induce phase separation by salting out. To remove any residual lipid content, solid-phase extraction columns and low-temperature freezing, where fipronil remains dissolved in the organic layer, but lipids solidify, or filters can be used prior to reconstitution with the evaporation of the solvent. The reconstitution achieves enrichment in accordance with the sensitivity of UHPLC-MS/MS analysis.

In the presented research, the fipronil extraction process was simplified to be more appropriate for on-site applications. We developed a complete, in-syringe, extraction method, which involves conducting the entire process inside a syringe, using only basic equipment. We eliminated sonication, head-over-head liquid–liquid extraction and centrifugation. The process consists of the following steps: an egg is cracked open, and the egg white is discarded in a waste container for biological material; to avoid homogenization, the egg yolk is retrieved with a syringe and the plunger is moved up and down to mix the yolk with the extraction solvent; and finally, the syringe is placed on a benchtop thermomixer to extract fipronil from the egg.

**Fig. 2:**
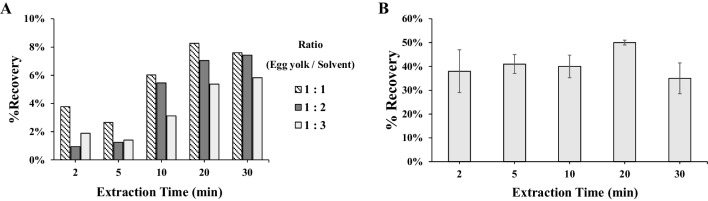
Fipronil extraction time optimization with respect to fipronil recovery by comparing the area of the UHPLC-MS/MS area for the *m/z* 435.0 > 330.0 transition of fipronil at different extraction times. **A** Spiked egg yolk (with fipronil 5 µg/kg, single measurement). **B** Spiked flower matrix (with fipronil 5 µg/kg, duplicate measurements). Error bars represent the standard deviation

Increased extraction recovery was obtained over time, reaching a plateau after 20 min (Fig. 2). Moreover, visually, the egg matrix precipitated better with a longer extraction time. Additionally, a higher egg-to-solvent ratio greatly affects the screening LFIA and the confirmatory UHPLC-MS/MS analysis, because it allows for higher fipronil recovery. Therefore, the optimized extraction time was chosen as 20 min, with a 1:1 v/v, egg yolk/methanol extraction ratio.

#### Fipronil extraction from sample of flowers

Compared to chicken eggs, flowers are a simpler matrix. To extract fipronil from flowers, a 1:1 w/v ratio of methanol was used. In a process similar to that of spiked egg yolk, spiked flowers were extracted for varying durations. Although longer extraction times resulted in darker extracts, there was no significant impact on fipronil recovery (Fig. 2). Therefore, a 5-min extraction time was selected as optimal.

### Smartcard design and fabrication

**Fig. 3:**
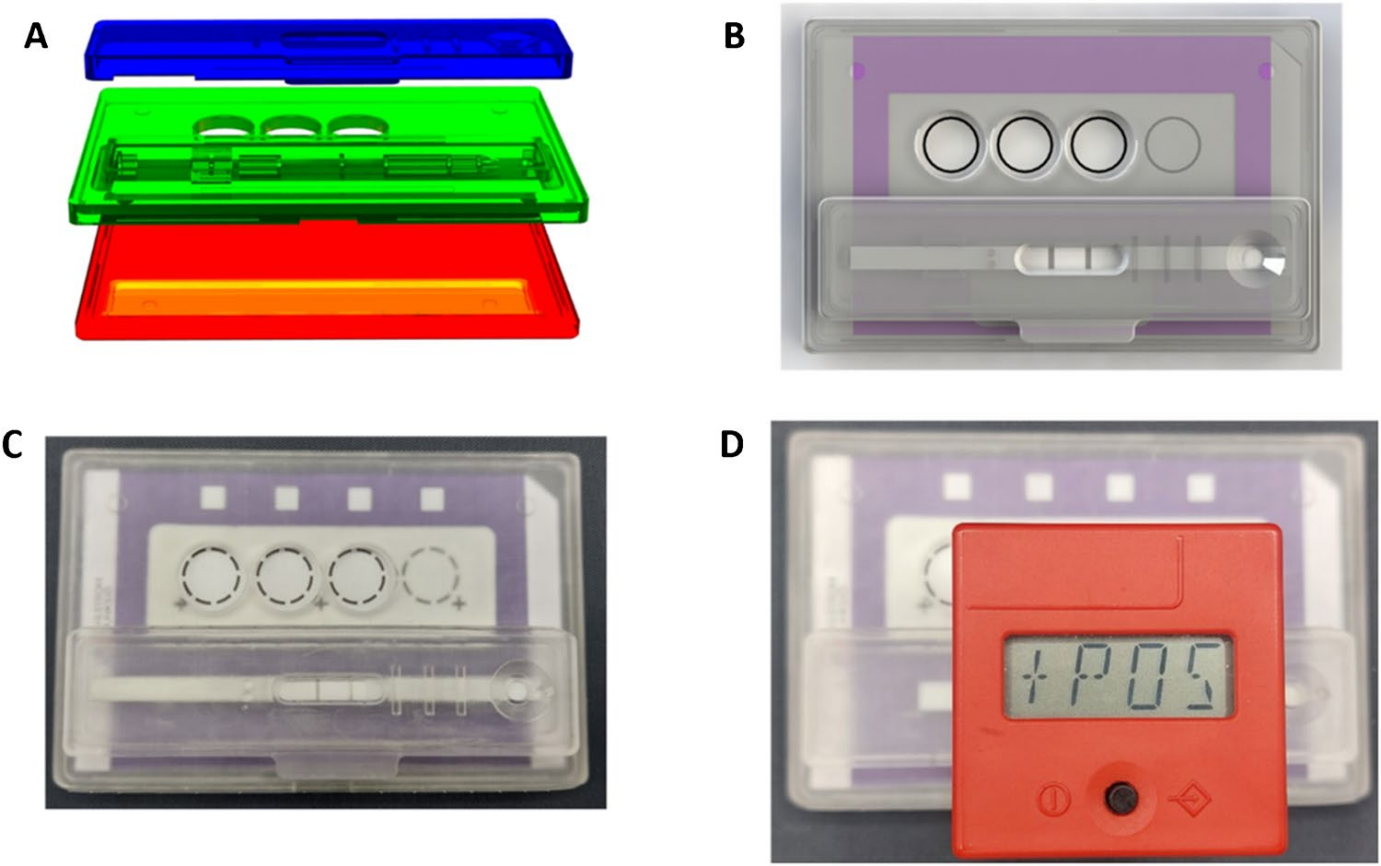
Overview of the Smartcard. **A** Exploded view of CAD models of the bottom part (red), top lid with LFIA cassette (green) and LFIA cassette lid (blue). **B** CAD model of the assembled Smartcard. **C** Photograph (top view) of the assembled Smartcard with DExS card and LFIA for fipronil. **D** Photograph (top view) of the assembled Smartcard with DExS card and the Cube Reader indicating a positive result from the LFIA development

The Smartcard (Fig. 3) has a compact size of 92 mm × 60 mm × 12 mm (*L* × *W* × *H*). It consists of three parts connected through bead and groove snap-fit joints. First, the bottom and top lid create a case for the DExS card. The bottom part is solid, allowing for secure positioning of the DExS card. In contrast, the top cover has three openings for easy and direct deposition of the extract on the DExS card and space for positioning the LFIA. The third part of the Smartcard is that of the LFIA cassette, which locks on the top lid, allowing for direct deposition of the diluted extract to develop the LFIA while providing the correct pressure points necessary for the optimum development of the LFIA. Additionally, the LFIA cassette features a protruding part that allows correct positioning of the Cube Reader for a semi-quantitative interpretation of the LFIAs. Short descriptions of the versions of the Smartcard and respective figures are presented in Supplementary Fig. 5.

### Screening LFIA for fipronil

#### Optimization and preparation

An icLFIA format was chosen to detect fipronil due to its suitability for low molecular weight analytes [49, 50]. In the icLFIA, the intensity of the test line is inversely proportional to the concentration of fipronil in the sample because the free fipronil from the sample extract competes for antibody binding with the fip-mAb on the LFIA. From the different combinations of fip-BSA and anti-fip mAb concentrations tested with the dotted LFIAs, the combination that led to the lowest visual detection amongst the tested was 500 µg/ml and 19.2 µg/ml, respectively, corresponding to a 10 µg/l fipronil concentration (Supplementary Fig. 6).

#### Sensitivity and preliminary evaluation

**Fig. 4:**
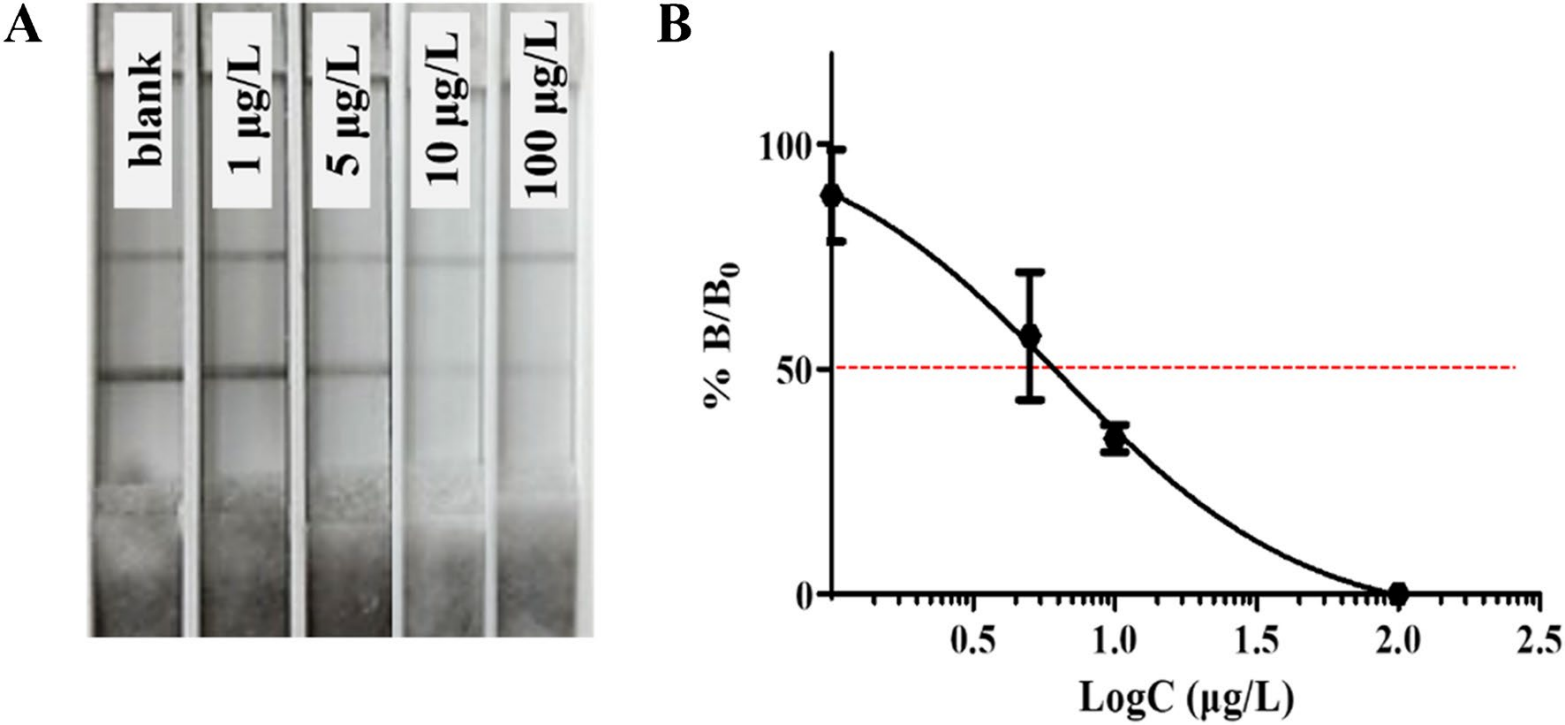
Sensitivity of the semi-quantitative LFIA (day 1 of preliminary evaluation). **A** Representative photos of the LFIA for fipronil demonstrating the fading of the test line with increasing concentration of fipronil. The photos presented are adjusted by a 10% increase in contrast and a 50% decrease in saturation. **B** Dose–response curve constructed using four-parameter logistic regression based on the average Cube readings of duplicate measurements. The IC_50_ (6.5 µg/l) is indicated by the red dotted line

The optimized LFIA was assembled as described in Supplementary Information. A range of calibration standards were loaded to the application zone to assess the inter-day variability of the fully assembled LFIAs. The results were visually assessed and measured using the Cube Reader, and the Cube results were plotted in dose–response curve (Fig. 4).

Based on the dose–response curve from day 1 of the preliminary evaluation, the half maximum inhibitory concentration (IC_50_) was determined to be 6.5 µg/l. Furthermore, after conducting one-way ANOVA of the preliminary inter-day variability evaluation over three consecutive days, it was found that there was no noteworthy distinction between the dose–response curves (Supplementary Fig. 7). This was supported by a statistical *P* value of 0.67, which indicates the null hypothesis cannot be rejected.

It is important to note that the optimized Smartcard approach involves diluting 20 µl of methanolic extract with 80 µl of running buffer to develop the screening LFIA. Theoretically, this dilution step lowers the final sensitivity by a factor of 5. Therefore, also the LFIA running buffer and Milli-Q water were examined as extraction solvents. More specifically, Milli-Q water was tested for its efficiency in extracting fipronil followed by dilution of 80 µl water extract with 20 µl 5 × concentrated running buffer. However, water alone did not sufficiently extract fipronil from the flowers, as indicated by the 40% sensitivity loss. Contrary, the running buffer–based extracts performed equally well to the four times diluted methanol extract for LFIA development (Supplementary Fig. 8); however, the LFIA buffer constituents are incompatible with MS instrumentation due to ion suppression that they may cause [51, 52].

### DExS card preparation and fipronil re-extraction

The DExS card has a dual function: (i) it simplifies transportation of sample extract by eliminating the need for bulky sample transfer and requires only a low-volume sample while (ii) improving analyte stability. Our approach involves depositing the methanolic sample extract onto the DExS card. Due to the absolute amount and the very low legal limit of detection for fipronil, a wax barrier was incorporated that enables higher extract volume deposition within a confined region of the card. After fipronil extraction, the sample is deposited onto the DExS card. Results showed that the pipetting position on the DExS card does not impact the final quantitation of the result (Supplementary Fig. 9).

**Fig. 5:**
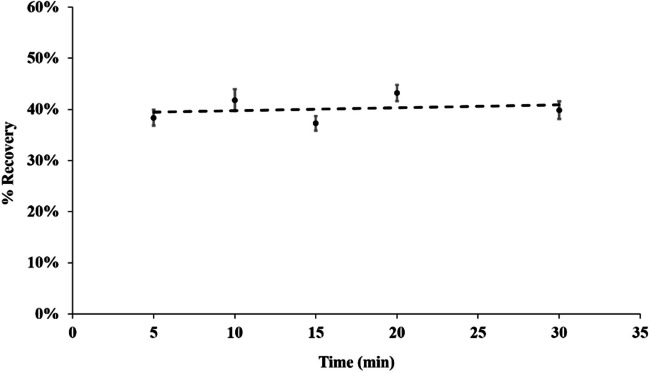
Fipronil extraction time optimization from DExS card. Recovery is calculated based on the ratio of signal (UHPLC-MS/MS area for the *m/z* 435.0 > 330.0 transition) of a 5 µg/kg fipronil standard solution spiked and recovered from the DExS card divided to a 5 µg/kg fipronil standard solution. The standard deviation of the duplicate measurement is shown by the error bars

To re-extract fipronil from the DExS card, a circular puncher (*Ø* 1 cm) was used to isolate a specific area (indicated by a dashed line on the card), and 100 µl methanol is used, which is the minimum required volume to ensure full submersion of the disc. To maximize the extraction of fipronil, agitation is carried out for 5 min. Although various extraction times were assessed, namely 5 min, 10 min, 20 min and 30 min, all resulted in approximately 40% recovery (Fig. 5). Thus, to facilitate a higher throughput approach, we ultimately chose the 5-min extraction.

In preparation for UHPLC-MS/MS analysis, the extract needs to be concentrated. This involves the removal of the solvent under a controlled N_2_ gas flow until complete evaporation is achieved. The resulting analyte is then reconstituted in 15 µl of methanol and subsequently analysed with UHPLC-MS/MS. It is crucial that the N_2_ flow gently contacts the surface of the solvent, without creating a disruptive vortex within it, which leads to loss of analyte. To evaluate the impact of different evaporation methods on standard solutions within a total recovery vial, a comparison was conducted between intense N_2_ evaporation, moderate N_2_ evaporation and overnight air drying of 5 µg/kg fipronil standard solutions in methanol. The results revealed no discernible difference between the sensitive N_2_ evaporation and the air-drying method. However, substantial losses were observed with the intense evaporation approach, amounting to approximately 87% (Supplementary Fig. 9).

Finally, flower extracts spiked with the target compound were applied to a DExS card, air dried and subsequently housed in a Smartcard at room temperature for a duration of 30 days. The ensuing UHPLC-MS/MS analysis, conducted in accordance with the comprehensive approach outlined in Fig. 1, proved successful in accurately quantifying the results across three distinct target levels (TLs), based on the maximum limit (ML) of 5 µg/kg specified in EU legislation, 2.5 µg/kg (0.5 × TL), 5 µg/kg (0.5 × TL) and 7.5 µg/kg (1.5 × TL). A paired *t* test was conducted to compare the concentration levels of the extracted samples stored for 30 days and those that are freshly extracted. The results indicated that there was no statistically significant difference in concentration levels between the two groups compared with a *P* value of 0.446. Therefore, we fail to reject the null hypothesis, suggesting that the concentration levels do not significantly differ between the two time points. This demonstrates the method’s capacity to preserve the DExS card for deferred analysis, offering flexibility in sample processing.

### UHPLC-MS/MS analysis

The applicability of the UHPLC-MS/MS method was demonstrated by detecting fipronil from spiked egg yolk at three different TLs based on the regulatory limit and blank. By following the optimized DExS card approach described in ‘General concept’, the area ion ratio of 69% (within the accepted deviation [53]) was estimated for all fipronil spiking levels of 2.5 µg/kg, 5 µg/kg and 7.5 µg/kg, i.e. 0.5 × TL, 1 × TL and 1.5 × TL, respectively, despite the low recovery (< 10%) from spiked egg yolk using methanol as extraction solvent. The UHPLC-MS/MS analysis of egg yolk spiked samples showed linearity with a linear regression coefficient of determination (*r*^2^) 0.98 and an LOD of 0.8 µg/kg.

### Smartcard approach in detecting fipronil contamination on flowers, from screening to lab-based confirmation

**Fig. 6:**
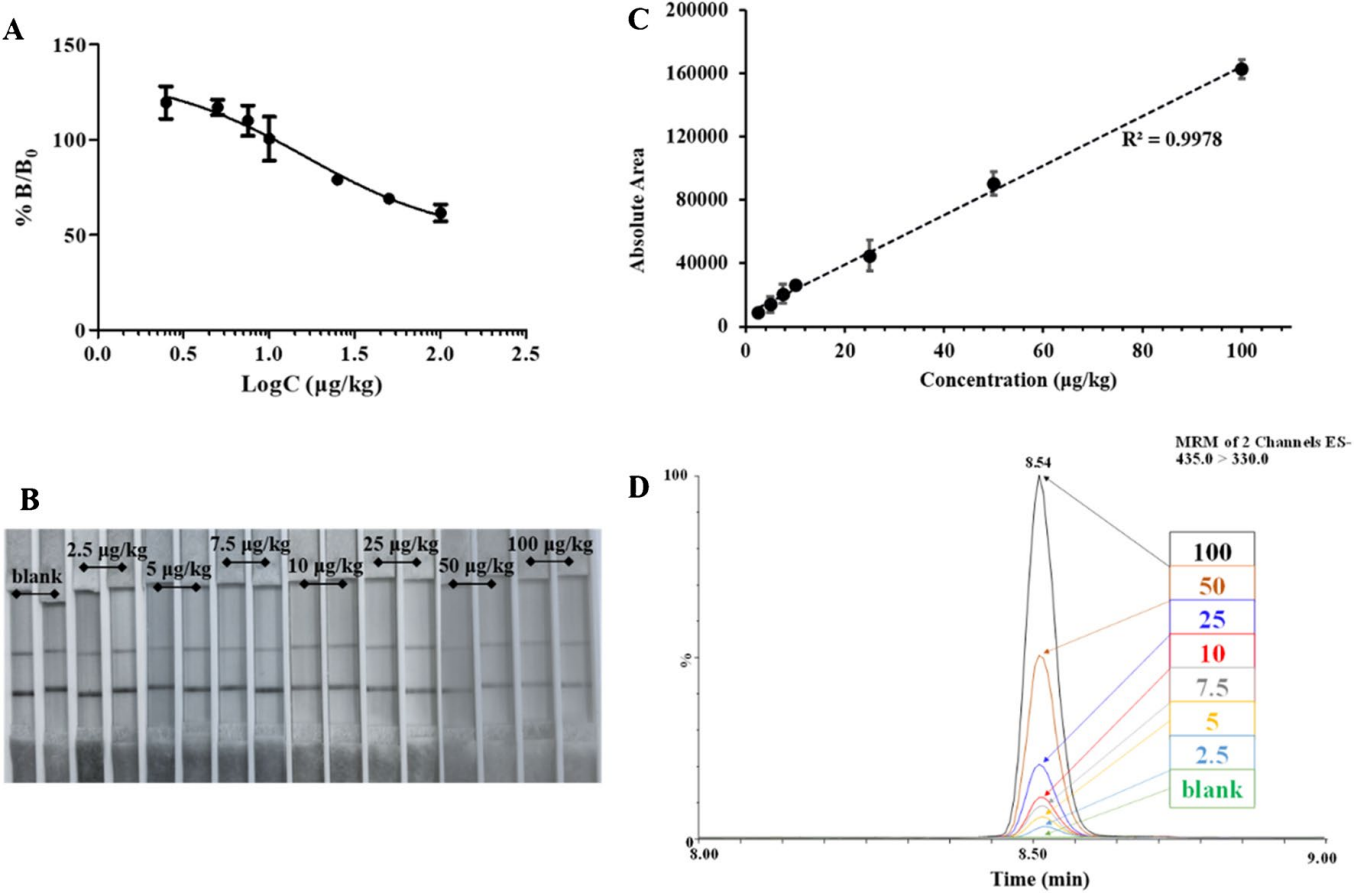
Analysis of fipronil spiked flowers, on different target levels, analysed with the complete Smartcard approach. **A** Dose–response curve from extracted fipronil-spiked flowers. **B** Screening LFIA optical results representative photos. The photos presented were adjusted by a 10% increase in contrast and a 50% decrease in saturation. **C** Respective UHPLC-MS/MS analysis calibration curve. **D** UHPLC-MS/MS overlay extracted ion chromatograms

The effectiveness of the integrated Smartcard method was demonstrated by testing duplicate spiked flower samples, i.e. two individual flowers spiked at 8 different levels ranging from 2.5 to 100 µg/kg, as well as a blank sample, extracted and tested by two LFIAs. UHPLC-MS/MS analysis showed excellent linearity with a linear regression coefficient of determination (*r*^2^) 0.998 and an LOD of 1.80 µg/kg. An ion ratio of 54% (within the accepted deviation [53]) was also estimated for all samples. The LFIA results showed an IC_50_ of 21 µg/kg based on the cube reading, and visually, a faded test line for concentrations over 10 µg/kg (Fig. 6). It is worth noting that the sensitivity of the fipronil LFIA system is heavily influenced by the bioreagents used, and lower IC_50_ and LOD values ranging from 0.73 to 4.89 µg/l and from 0.14 to 3.69 µg/l, respectively, have been cited in the literature in other systems [49, 54]. So, the current approach could be used for a risk-based sampling and a rapid on-site assessment of the fipronil contamination in different commodities before official confirmatory UHPLC-MS/MS analysis, due to the use of the Smartcard.

## Conclusions

In the presented research, we demonstrated the conceptualization, development and optimization of a methodical approach that bridges on-site sampling and screening with high-end instrumental analysis. Our approach involves a Smartcard integrating a wax-treated dried extract spot (DExS) card and a dedicated LFIA. After a simplified in-syringe on-site extraction process, the LFIA is developed, and the extract is deposited on the DExS card. The Smartcard is then transported to a routine analysis lab, and the targeted contaminant is re-extracted and analysed with UHPLC-MS/MS for confirmatory analysis. We have successfully introduced this method to analyse fipronil contamination on flowers and in eggs, highlighting the importance of reliable and efficient on-site screening methods for monitoring contaminant residues in food. This sampling and analysis approach shows great potential for quick screening of food commodities for high throughput screening, and risk-guided sampling for confirming contamination in the laboratory, thus contributing in enhancing food safety and ensuring quality in the food supply chain. Further improvement in the approach could be achieved by incorporating more suitable instrumentation for on-site use. For instance, replacing the thermomixer, which ensured repeatability during development, with more portable alternatives such as vortex mixers, manual shaking or battery-powered magnetic stirrers. Moreover, moving towards more sustainable options, such as biodegradable plastic, could help mitigate potential negative environmental impact of the Smartcard.

Additionally, the screening LFIA is less sensitive for the real sample application due to the necessary dilution factor for development. The choice of methanol as extraction solvent, despite being necessary for the extraction efficiency, still remains a suboptimal choice for on-site applications and use from non-trained personnel. Further approaches could include testing more complex systems that incorporate a pre-concentration sample pad and eliminate the use of methanol. Lastly, implementing a robot for automatic processing in the lab [55] of the DExS card in UHPLC-MS/MS analysis could reduce the required manual labour and increase the throughput capabilities in routine analysis laboratories. Finally, including the targeted analysis of fipronil metabolites for application in the LFIA and the extraction process could provide a comprehensive analysis of total fipronil contamination.

## Supplementary Information

Below is the link to the electronic supplementary material.

Supplementary file1 (DOCX 2.35 MB)
