# Supplementary material for: Smartcard: an integrated approach for contaminant monitoring, from field to laboratory
Source: Anal Bioanal Chem. 2024 Nov 12;417(1):69–82. doi: 10.1007/s00216-024-05626-w (PMC11695648; doi:10.1007/s00216-024-05626-w)
Supplement: Supplementary file 1 — Supplementary file1 (DOCX 2.35 MB) [file 216_2024_5626_MOESM1_ESM.docx]

**Supplementary information**

**Smartcard; an integrated approach for contaminant monitoring, from field to laboratory**

Ariadni Geballa-Koukoula^1^, Linda Willemsen^1^, Erik Beij^1^, Richard van Hoof^1^, Alexander Elferink^1^, Khalil Geballa-Koukoulas^2^, Jeroen Peters^1^, Marco H. Blokland^1^ and Gert IJ. Salentijn^1,3*^

^1^ Wageningen Food Safety Research, Wageningen University and Research, P.O. Box 230, 6700 AE Wageningen, The Netherlands

^2^ Laboratory of General and Agricultural Microbiology, Department of Crop Science, Agricultural University of Athens, Iera Odos 75, 118 55 Athens, Greece

^3^ Laboratory of Organic Chemistry, Wageningen University, Stippeneng 4, 6708 WE Wageningen, The Netherlands

* gert.salentijn@wur.nl

**Table of content**

1. Material and methodsS3

1.1. Assembly of screening LFIA for fipronil S3

Figure S1. Schematic of the complete fipronil icLFIA S4

Figure S2. Optical evaluation for result interpretation of the icLFIA for fipronil S4

1.2. DExS card preparation S5

Figure S3. Preparation of the DExS card S5

2. Results S5

2.1. Extraction solvent selection S5

Table S1. Compatibility studies of solvents S5

Figure S4. Compatibility testing results for the LFIA developedS6

2.2. Smartcard design and fabricationS6

Figure S5. CAD designs of Smartcard versionsS7

2.3. Screening LFIA optimization S7

2.3.1. Optimization and preparation S7

Figure S6. Determination of the optimum test spot S7

3.3.2. Sensitivity and preliminary evaluation S8

Figure S7. Preliminary inter-day variability results S8

Figure S8. Comparison of LFIA Cube readings of spiked flower extracts of different solutions S8

2.4. DExS card fipronil extraction optimization S9

Figure S9. DExS card fipronil re-extraction optimization S9

1. **Material and methods**
   1. **Assembly of screening LFIA for fipronil**

For the assembly of the LFIA for fipronil, the CNP-GaM conjugate was diluted 10-fold in the spraying buffer, and 750 μL were pipetted onto a 30 cm x 0.4 cm glass fiber pad, and dried overnight. Similarly, an antibody pad was used, where anti-fip monoclonal antibodies (mAb) at an optimized concentration of 19.2 μg/mL were further diluted 10-fold in the same spraying buffer, and 750 μL were pipetted on a separate 30 cm x 0.4 cm length glass fiber. Both pads were allowed to air dry overnight.

During assembly, the nitrocellulose membrane containing the C and T lines was securely attached in the middle of a 6 cm plastic backing support, and a 2 cm absorbent pad was positioned at the end of the nitrocellulose, overlapping by 2 millimetres. The CNP-GaM glass fiber pad was then secured on the plastic backing support at a distance of 1 cm from the beginning of the backing support, followed by the anti-fip mAb glass fiber pad, which overlapped the nitrocellulose by 1 mm. Finally, both pads were overlaid with a 2 cm sample pad.

After full assembly, the LFIA 30 cm card was cut into 4 mm-wide strips using a guillotine and placed (**Figure S1**).

| 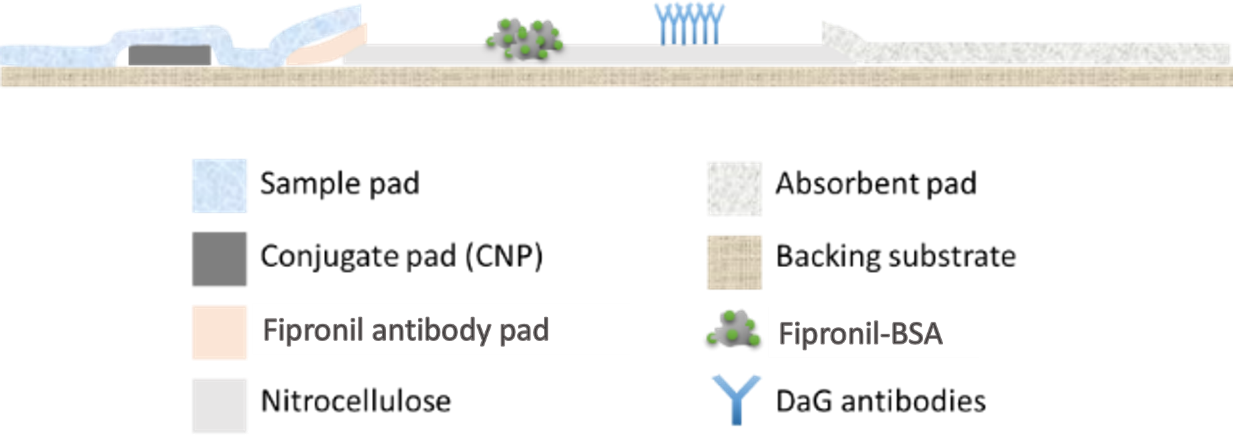 |
| --- |
| **Figure S1. Schematic of the complete fipronil icLFIA presenting the different biomolecules and components used. Fipronil-BSA indicates the test line position, and DaG pAb indicates the control line.** |

| 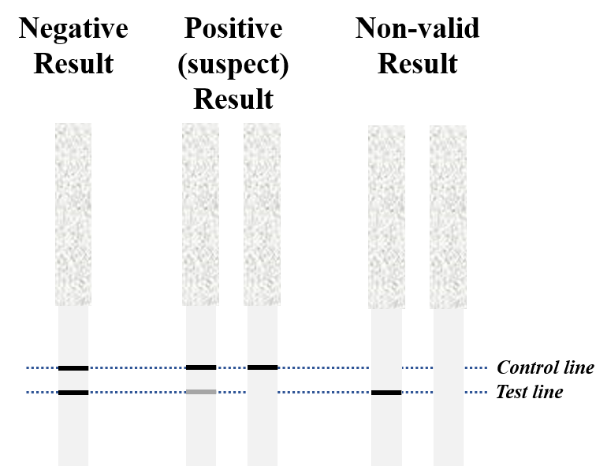 |
| --- |
| **Figure S2. Optical evaluation for result interpretation of the icLFIA for fipronil. Due to the competitive principle of the LFIA, two lines indicate a screening negative result, whereas faint or absent test line indicate fipronil presence in the sample. Absense of the control line indicates a non-valid screening result. that needs to be repeated.** |

- 1. **DExS card preparation**

| 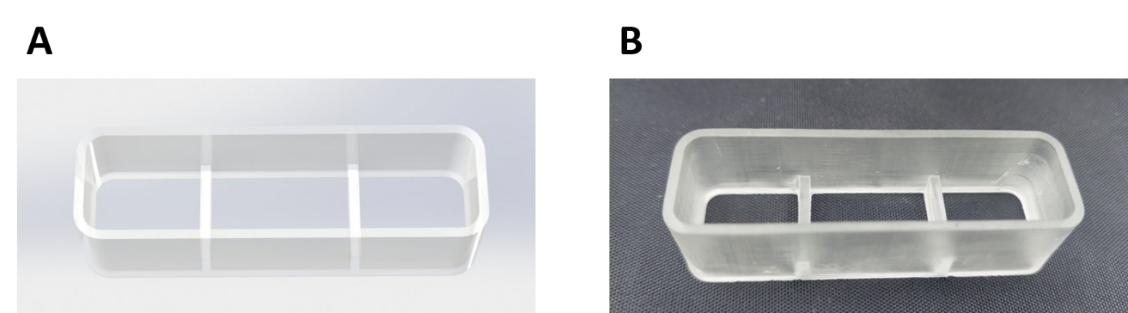 |
| --- |
| 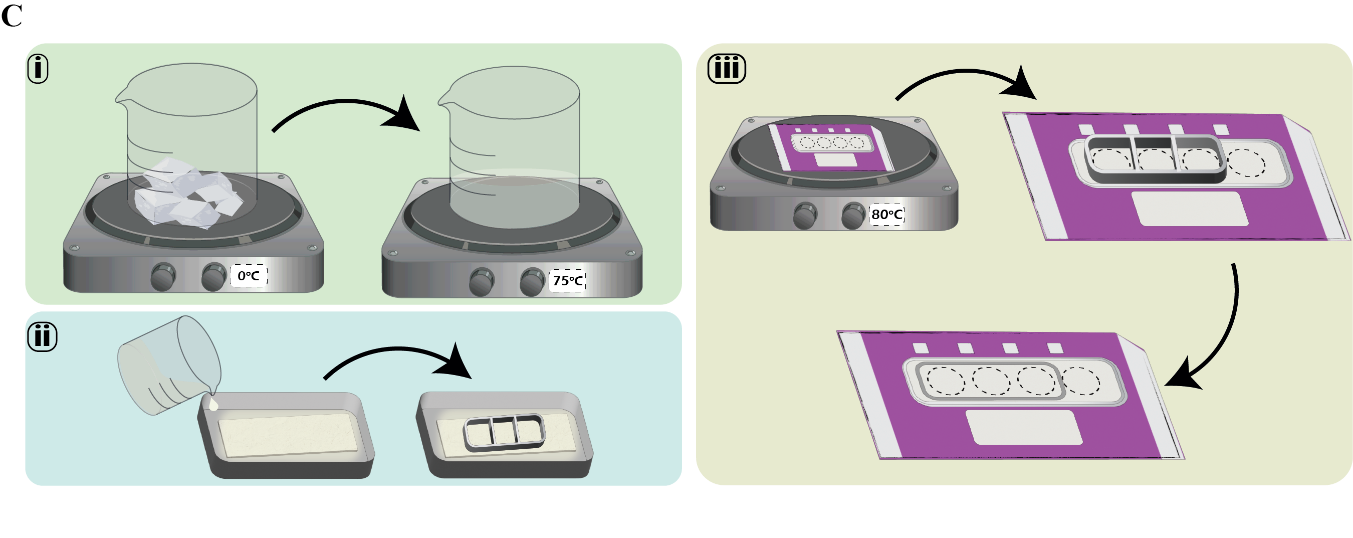 |
| **Figure S3. Preparation of the DExS cards; (A) CAD design and (B)real-life photo of the 3D-printed wax stamp used to prepare the DExS cards, and (C) step-by-step schematic illustration of the stamping process; i. Paraffin wax is melted at 75^o^C, ii. The paraffin wax is poured on top of a cellulose pad to create a stamp pad, and the 3D-printed stamp is pressed onto the stamp pad, iii. Pre-heated DBS card at 80^o^C for 1min, and stamped onto the DBS card, resulting in the DExS card.** |

1. **Results**
   1. **Extraction solvent selection**

| **Table S1. Compatibility studies of solvents for 3D-printing resins, LFIA and egg yolk.** | | | |
| --- | --- | --- | --- |
| **Solvent** | **3D-printing material weight gain** | **LFIA*** | **Egg yolk** |
| Water | 0.21% | Compatible | Compatible |
| Methanol | 0.57% | Compatible | Compatible |
| Acetonitrile | 0.25% | Not compatible | Compatible |
| Acetone | Cracking | Not compatible** | Not compatible |
| Ethyl acetate | 0.53% | Not compatible | Not compatible |
| Ethanol | 0.35% | Not compatible | Compatible |
| Acetonitrile/formic acid 1% *v/v* | 0.25% | Not compatible | Compatible |
| * Refers to 20% v/v organic solvent in running buffer. As not compatible are characterised the solvents causing false positive results when the LFIA is developed with 20% v/v organic solvent in running buffer (blank). **Acetone dissolves the LFIA nitrocellulose. | | | |

| **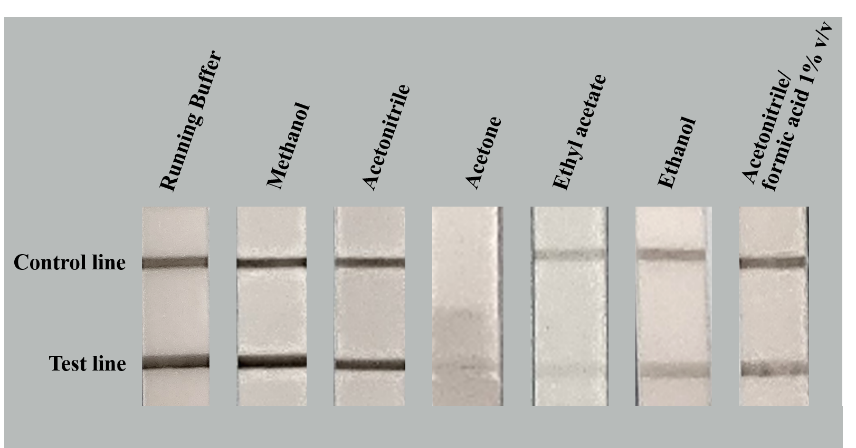** |
| --- |
| **Figure S4. Compatibility testing results for the LFIA developed in 20% v/v organic solvent in running buffer. Only blanks were tested. LFIA constituents: 0.15 mg/ml DaG (control line), 1  mg/ml fipronil-BSA (test line), running buffer: 0.01M PBS + 1%w/v BSA + 0.05% v/v tween-20. anti-fip mAb were diluted in 0.01M PBS. The LFIAs were developed in a 96-well plate. In each well the following were added: 1 µl diluted antibody anti-fip mAb, 1 µl GaM-CNP 98 µl running buffer containing 20% v/v organic solvent. Results were assessed after 10 minutes of development.** |

- 1. **Smartcard design and fabrication**

Several variations of the Smartcard were created to accommodate both the LFIA cassette and the DExS card. The initial design utilized supermagnetes to secure the LFIA cassette lid, as well as a case cover. However, this version was somewhat bulky, and the magnets caused the casing to cling to metallic objects. Furthermore, the DExS card was not securely held in place, increasing the risk of loss. A revised version was developed that utilized protruding notches to hold the card in place, eliminating the need for the bulky case cover and reducing the overall size of the holder. The final version incorporated snap-fit joints, allowing for easy opening and a snug fit for the DExS card. The use of snap-fit joints also eliminated the need for magnets in the LFIA cassette and ensured proper pressure points.

| 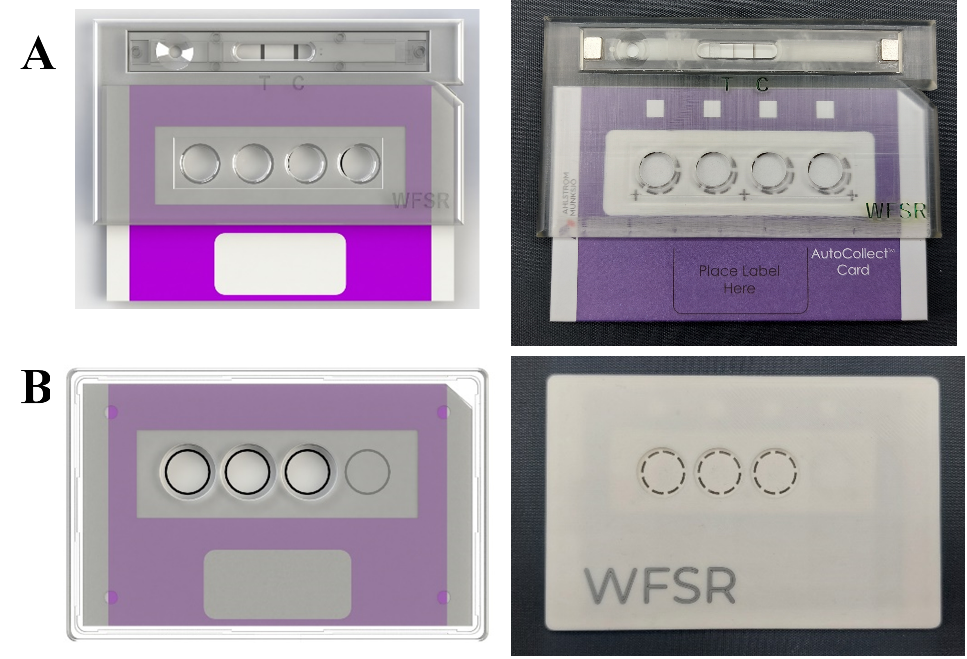 |
| --- |
| **Figure S5. CAD designs (left) and real-life images (right) of the different versions of the Smartcard designed. A) The first bulky design with supermagnets, and B) the second improved design with snap-fit joints to hold the Smartcard in place.** |

- 1. **Screening LFIA optimization**
     1. **Optimization and preparation**

| **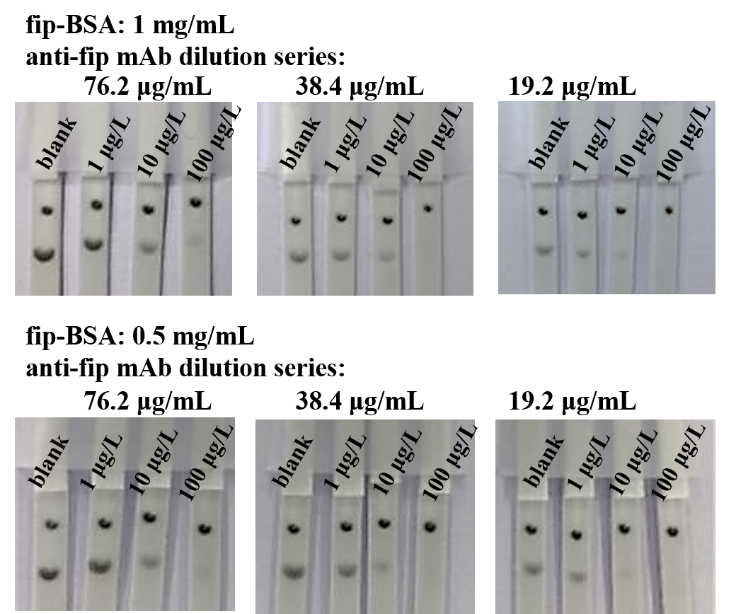** |
| --- |
| **Figure S6. Determination of the optimum test spot (fip-BSA) and anti-fip mAb concentrations using spotted icLFIAs. The test included calibration standards of fipronil in running buffer, at 1, 10, 100 μg/L and blank (0 μg/L), to assess the optimal sensitivity possible based on clear visual detection by the naked eye.** |

- - 1. **Sensitivity and preliminary evaluation**

| 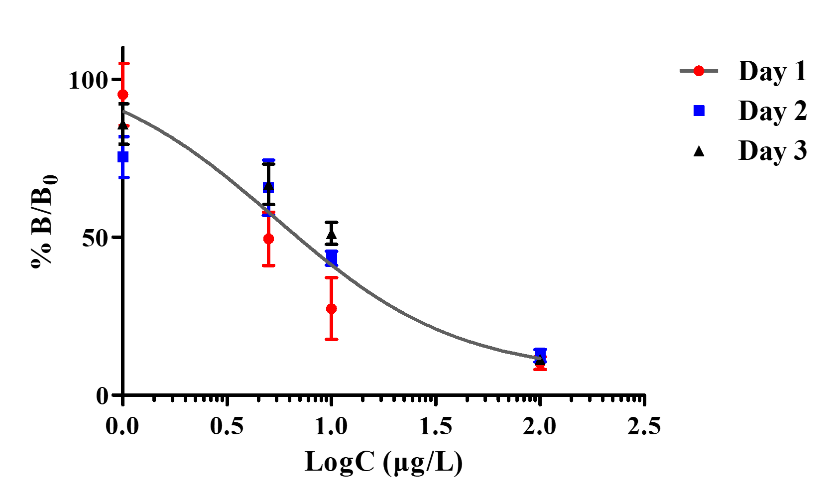 |
| --- |
| **Figure S7. Preliminary inter-day variability expressed by dose-response curve constructed by four-parameters logistic regression, based on the average Cube readings (n=2) of duplicate measurements over three different days.** |

|  |
| --- |
| **Figure S8. Bar graph comparison of LFIA average Cube readings (n=2) of spiked flower extracts of different solutions (Milli-Q water, running buffer, and methanol) for different spiking concentrations (10 and 100μg/kg) and blank. Results indicate minimal differences between blank and highly spiked samples for water extraction, signifying its insufficient for extracting fipronil from spiked flowers. Contrary, buffer, and methanol showed similar performance after extraction, resulting in weaker intensities of the test line for spiked samples.** |

- 1. **DExS card fipronil extraction optimization**

| 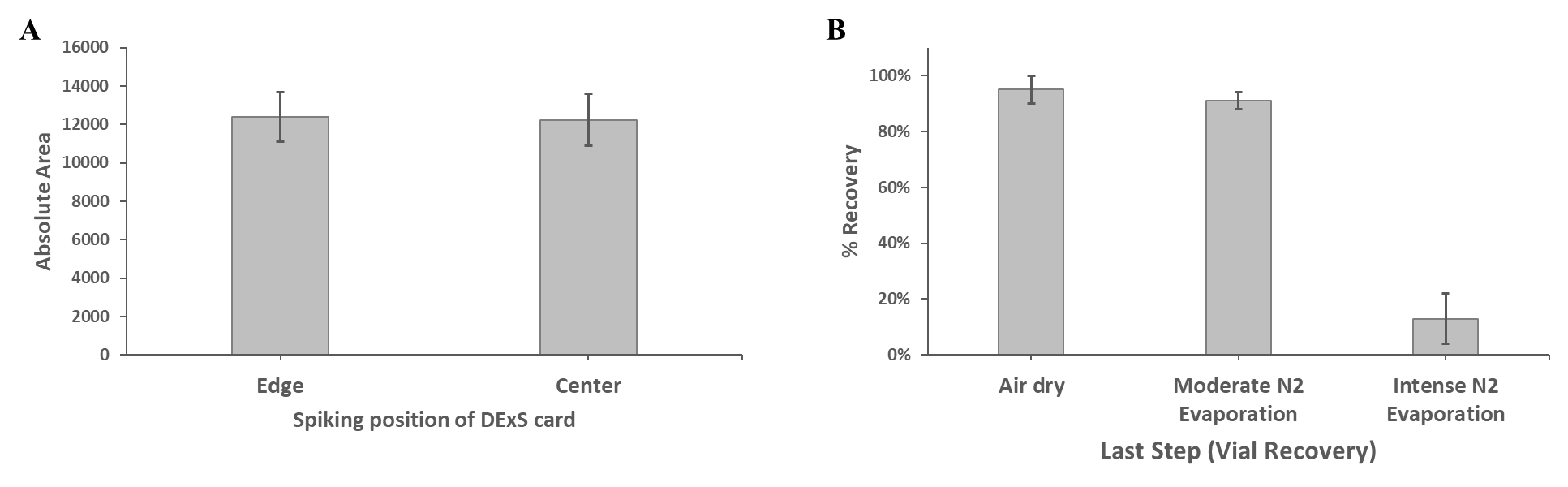 |
| --- |
| **Figure S9. DExS card fipronil re-extraction optimization by comparison of the UHPLC-MS/MS area for the m/z 435.0 > 330.0 transition of fipronil (5 μg/kg fipronil standard solution). A) fipronil recovery from pipetting solution on different parts of the DExS card, i.e., the edge or the centre, as those parts indicated by the dashed line on the card, indicating no difference on the quantitation of the results from the different pipetting site B) solvent evaporation optimization required for the concentration step preceding the UHPLC-MS/MS analysis of the Smartcard approach. Results indicate that moderate to no N_2_ flow evaporation should be used to ensure minimum sample loss.**  **The standard deviation of the duplicate measurement is shown by the error bars.** |
